# Tuning the Structural, Acidic, and Catalytic Properties of SAPO-11 by Varying the SiO_2_/Al_2_O_3_ Ratio in a Boehmite-Based Reaction Gel

**DOI:** 10.3390/gels11120989

**Published:** 2025-12-08

**Authors:** Arthur R. Zabirov, Dmitry V. Serebrennikov, Nadezhda A. Filippova, Denis Sh. Sabirov, Arthur I. Malunov, Ekaterina S. Mescheryakova, Rufina A. Zilberg, Marat R. Agliullin

**Affiliations:** 1 Institute of Petrochemistry and Catalysis, Ufa Federal Research Centre of the Russian Academy of Sciences, 450075 Ufa, Russia; best535@inbox.ru (A.R.Z.); d25c25@yandex.ru (D.V.S.); diozno@mail.ru (D.S.S.); malunovv@mail.ru (A.I.M.); katusha2974@gmail.com (E.S.M.); maratradikovich@mail.ru (M.R.A.); 2 Department of Analytical Chemistry, Ufa University of Science and Technology, 450076 Ufa, Russia; zilbergra@yandex.ru

**Keywords:** molecular sieves, silicoaluminophosphate SAPO-11, reaction gels, nanoscale crystals, 1D channels, hydroisomerization of *n*-C_16_

## Abstract

The rational design of highly efficient bifunctional SAPO-11 catalysts for hydroisomerization of *n*-C_16_ requires unprecedented control over both acidic properties and diffusion characteristics. This work systematically investigates the influence of the SiO_2_/Al_2_O_3_ molar ratio (0.1–0.4) in the initial gel on the physicochemical and catalytic properties of SAPO-11. Using a combination of characterization techniques (XRD, SEM, TEM-SAED, ^29^Si MAS NMR, and IR-Py), it was established that this parameter serves as a simple tool for crystal engineering. The concentration of Brønsted acid sites and the external surface area demonstrate a non-linear dependency, reaching their maximum at SiO_2_/Al_2_O_3_ = 0.3. Further increase in silicon content reduces both crystallinity and acidity due to the transition to the dominant SM2 + SM3 incorporation mechanism and the formation of silicon islands. Notably, varying the SiO_2_/Al_2_O_3_ ratio enables control over crystal morphology—progressing systematically from truncated cones (SiO_2_/Al_2_O_3_ = 0.1) to flat prismatic platelets (SiO_2_/Al_2_O_3_ = 0.2) and ultimately hierarchical spherical aggregates (SiO_2_/Al_2_O_3_ = 0.4). In *n*-C_16_ hydroisomerization, the Pt/SAPO-11(0.2) catalyst demonstrated the highest yield of *i*-C_16_ compared to other samples reaching 81%. The platelet morphology ensures a minimal diffusion path (<100 nm), effectively suppressing secondary hydrocracking. This finding underscores that morphology optimization is more critical than maximizing acidity for achieving high selectivity in the context of *n*-C_16_ hydroisomerization over Pt/SAPO-11.

## 1. Introduction

Silicoaluminophosphate (SAPO-n) molecular sieves, first reported by Wilson et al. in 1984 [1], have rapidly established themselves as the second most significant class of zeolitic materials. Their unique combination of structural and acidic properties forms the basis for large-tonnage industrial processes, such as the methanol-to-olefins (MTO) conversion over SAPO-34 [2] and the catalytic isodewaxing of diesel fuels over SAPO-11 [3]. Within this family, the SAPO-11 molecular sieve (structural type AEL) is of particular interest. Its unique one-dimensional channel structure, composed of elliptical pores measuring 4.0 × 6.5 Å [4], is ideally suited for implementing the principles of shape-selective catalysis [5,6]. This makes bifunctional catalysts based on it highly selective in key oil refining reactions, primarily in the hydroisomerization of higher *n*-paraffins (C_16+_) for the production of low-pour-point fuels and base oils [3,7,8,9], as well as in a number of other processes [10,11].

The catalytic properties of SAPO-n materials are determined by their Brønsted acid sites (BAS), which are formed through the isomorphic incorporation of silicon into the neutral aluminophosphate framework during hydrothermal crystallization. A key feature of this process, which distinguishes SAPO-n from classical zeolites, is the possibility of Si incorporation via two distinct substitution mechanisms (SM): SM2 and SM2+SM3 [12,13]. The SM2 mechanism (“single” substitution of Si for P) leads to the formation of isolated Si–O(H)–Al bridges, generating BAS of moderate strength. In contrast, the SM2+SM3 mechanism involves the incorporation of silicon in the form of “silicon islands” (SiO_2_), which are believed to generate stronger BAS. Thus, controlling the ratio between these mechanisms is a fundamental tool for the fine-tuning of both the concentration and the strength of the acid sites.

Current research aimed at enhancing the efficiency of SAPO-11 is focused on addressing two key challenges. The first is the reduction in diffusion limitations for bulky C_16+_ *n*-paraffin molecules through the synthesis of nanosized crystals or hierarchical pore systems [14,15,16,17,18]. The second, equally important challenge, is the control of the aforementioned acidic properties. It is known that the mechanism of silicon incorporation can be governed by varying the parameters of the reaction gel and the crystallization process, including the nature of the template [19,20], the type of silicon source [21,22], and the use of organic solvents or two-phase systems [18,23,24].

Thus, our previous studies [25,26] demonstrated that using a SiO_2_ sol with an average particle size of 4 nm as the silicon source enabled the synthesis of a silicoaluminophosphate SAPO-11 with a high concentration of strong Brønsted acid sites (BASs), as well as with varied crystal morphology and size. However, despite these findings and existing data on the influence of the silicon source type, one of the most fundamental parameters—the initial SiO_2_/Al_2_O_3_ molar ratio in the reaction gel—and its comprehensive impact on the properties of the final material remain insufficiently studied. The literature provides only isolated indications, for example, of a saturation in acid site concentration upon reaching an SiO_2_/Al_2_O_3_ ratio > 0.5 [27]. Furthermore, it is practically unexplored how this parameter simultaneously influences not only acidity but also other critically important characteristics: phase purity, crystal morphology, and size. These structural and morphological parameters, in turn, have a direct impact on the diffusion properties and, consequently, on the overall catalytic performance in C_16+_ hydroisomerization.

The present work is devoted to a systematic investigation of the effect of the SiO_2_/Al_2_O_3_ molar ratio in the initial synthesis gel on the physicochemical properties of SAPO-11 (crystallinity, morphology, particle size, distribution and strength of acid sites) and to establishing correlations between these characteristics and the catalytic performance of the resulting materials in the hydroisomerization of *n*-hexadecane.

## 2. Results and Discussion

The silicon content in the SAPO-n framework is a key factor determining its acidic and, consequently, catalytic properties [28,29]. In this regard, we investigated how the composition of the initial reaction gel affects the final silicon content in the crystalline products (Table 1). It was found that the silicon content in the final products is consistently lower than in the initial gels, indicating incomplete incorporation of Si during the crystallization process. Notably, when the SiO_2_/Al_2_O_3_ molar ratio in the gel exceeds 0.3, no further increase in the silicon content within the SAPO-11 crystalline framework is observed. This effect appears to be related to reaching the incorporation limit of silicon into the AEL aluminophosphate structure. The excess silicon likely remains in the mother liquor or forms a separate amorphous phase.

**Figure 1:**
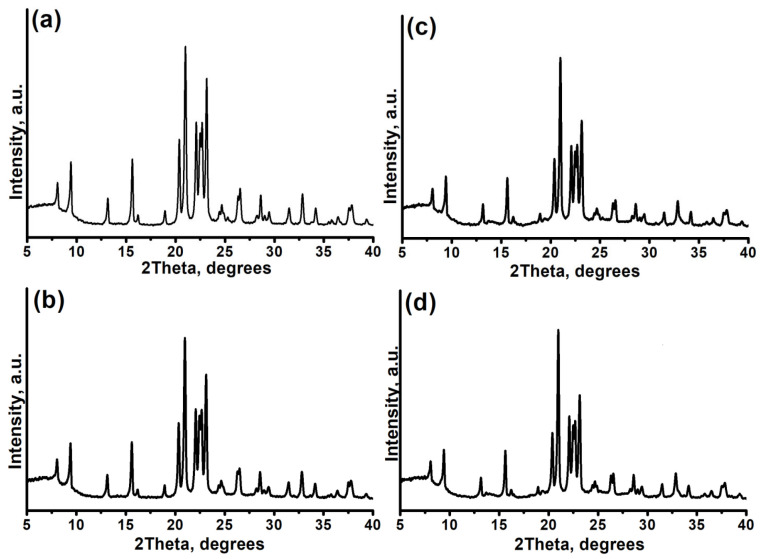
X-ray diffraction patterns of crystalline silicoaluminophosphates synthesized at different SiO_2_/Al_2_O_3_ ratios: (**a**) SAPO-11-0.1; (**b**) Sample SAPO-11-0.2; (**c**) Sample SAPO-11-0.3, (**d**) Sample SAPO-11-0.4.

Furthermore, the phase purity and crystallinity of the synthesized materials were evaluated by powder X-ray diffraction (PXRD) (Figure 1). All synthesized samples exhibit diffraction patterns characteristic of the AEL structural type (PDF № 00-041-0023) and possess high phase purity. However, a clear trend of decreasing relative crystallinity degree is observed with an increase in the silicon content in the initial gel. In particular, at an SiO_2_/Al_2_O_3_ ratio > 0.4, the crystallinity degree does not exceed 81%. This decline is likely due to two factors. Firstly, excess silicon, not incorporated into the framework, may be present as an X-ray amorphous SiO_2_ phase. Secondly, it is well-known for SAPO-11 [30] that silicon can incorporate not only via the isomorphic substitution of P by Si(4Al) but also by forming so-called “silicon islands” (Si-O-Si) [12]. Such formations disrupt the regularity of the aluminophosphate framework, leading to the formation of structural defects and, consequently, a reduction in overall crystallinity.

**Figure 2:**
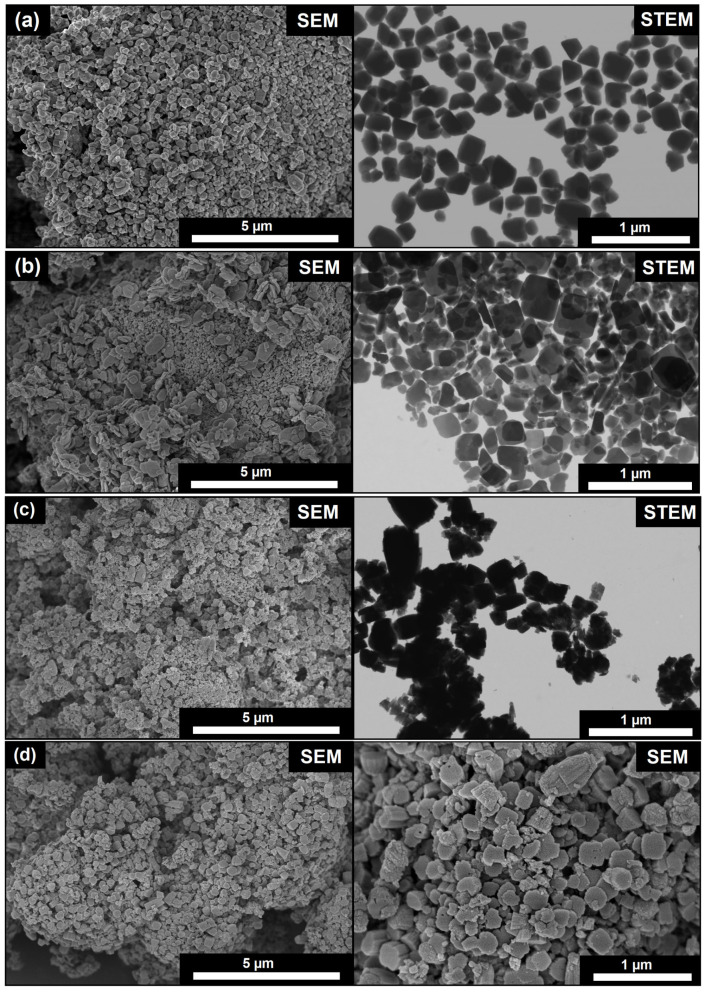
SEM micrographs of SAPO-11 samples synthesized at different SiO_2_/Al_2_O_3_ ratios: (**a**) SAPO-11-0.1; (**b**) SAPO-11-0.2; (**c**) SAPO-11-0.3; (**d**) SAPO-11-0.4.

In addition to influencing the composition and crystallinity, the silicon content in the gel has a strong effect on crystal morphology and size. This aspect is critical because the morphology and crystal size determine both the external surface area and the diffusion path length to the active sites. Scanning electron microscopy (SEM) (Figure 2) was used to study this relationship. SEM and, particularly, STEM imaging reveal a clear trend in crystal size distribution across the series. As the SiO_2_/Al_2_O_3_ ratio increases from 0.1 to 0.4, the uniformity of SAPO-11 crystals decreases, and the particle size distribution becomes progressively broader. This widening is most pronounced for the SAPO-11(0.4) sample, which exhibits significant heterogeneity in crystal dimensions, in contrast to the relatively narrow size distribution observed for SAPO-11(0.2). The obtained SEM data revealed a correlation between the Si content and morphology: SAPO-11(0.1) (SiO_2_/Al_2_O_3_ = 0.1) consists of uniform crystals in the form of truncated cones, 200–300 nm in average size. Increasing the SiO_2_/Al_2_O_3_ ratio to 0.2 (SAPO-11(0.2)) leads to a change in morphology: flat prisms (platelets) with a length of 200–400 nm and a thickness of about 80 nm are formed. A further increase in the SiO_2_/Al_2_O_3_ ratio to 0.3 (SAPO-11(0.3)) leads to the formation of irregularly shaped intergrowths, which appear to consist of a mixture of crystals of various habits (cones, prisms, cubic particles) sized 100–300 nm. In contrast, SAPO-11(0.4) (SiO_2_/Al_2_O_3_ = 0.4) forms large hierarchical spherical aggregates, 3–5 μm in size. These spheres are composed of densely packed nanocrystals in the form of cones (200–400 nm), oriented with their vertices toward the center of the aggregate. Thus, the SEM data show that varying the silicon concentration is a simple tool for controlling the crystal morphology of SAPO-11, ranging from discrete nanocrystals to complex hierarchical nanostructures.

**Figure 3:**
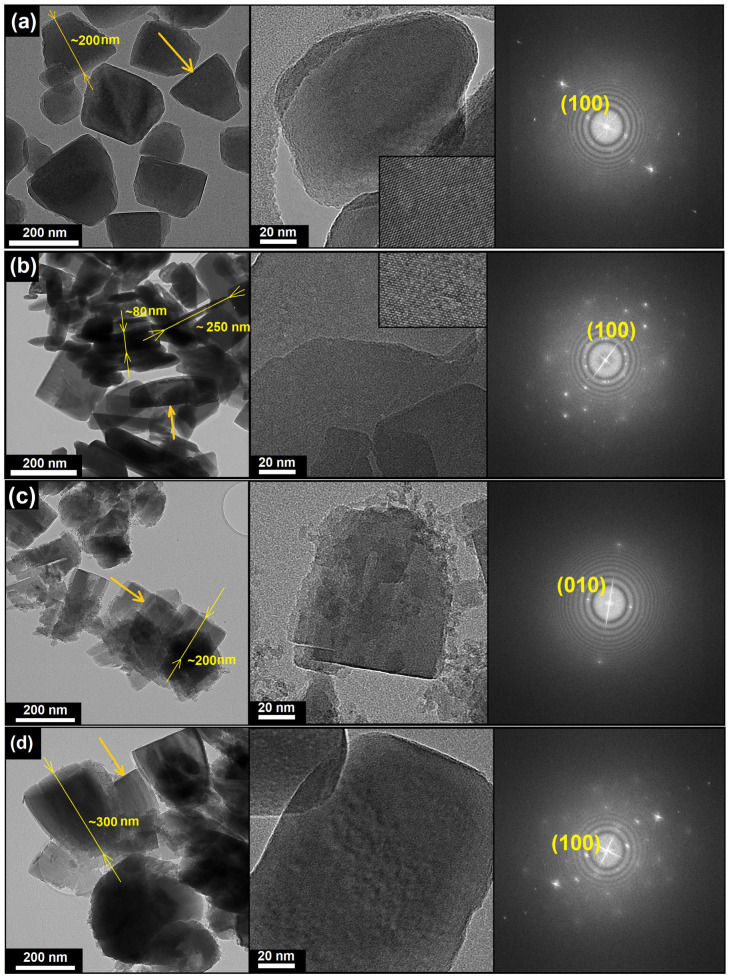
TEM micrographs and corresponding SAED patterns of SAPO-11 synthesized at different SiO_2_/Al_2_O_3_ ratios: (**a**) SAPO-11-0.1; (**b**) SAPO-11-0.2; (**c**) SAPO-11-0.3; (**d**) SAPO-11-0.4. The arrow in the image indicates the location where the diffraction pattern was obtained.

The varying crystal morphology may imply different orientations and lengths of the internal 1D 10-ring (10R) channels, which directly affects the molecular sieve’s diffusion properties. To determine the orientation of the 1D 10R channels in samples with different morphologies, transmission electron microscopy (TEM) and Fast Fourier Transform (FFT) pattern analysis were employed (Figure 3). For the SAPO-11(0.1) sample (truncated cones), the FFT analysis showed a diffraction pattern typical of a single crystal. The determined interplanar spacing of d = 1.32 nm corresponds to the (100) plane (theoretical d = 1.34 nm). This indicates that the one-dimensional channels are oriented along the cone’s axis. TEM images (Figure 3) confirm this, showing the channel mouths on the base of the cone. Consequently, the diffusion path length in this sample is equivalent to the cone’s length and is approximately 200 nm. A similar analysis for SAPO-11(0.2) (flat prisms) also revealed the dominance of the (100) plane on the crystal’s primary (most developed) surface. This means that the 1D 10R channels in these crystals are aligned along the prism’s thinnest axis. Therefore, the channel length (diffusion path) in this sample is minimal and is less than 100 nm (corresponding to the platelet’s thickness).

In contrast, the analysis of a crystal with a cubic habit from the multimodal SAPO-11(0.3) sample showed a different orientation. The interplanar spacing of d = 0.942 nm on the side faces corresponds to the (010) plane (theoretical d = 0.935 nm). This is the classic orientation for SAPO-11, where the channels run along the prism’s long axis (~300 nm).

Finally, the hierarchical SAPO-11(0.4) sample, composed of intergrown cones, demonstrated the same orientation as SAPO-11(0.1): d = 1.32 nm ((100) plane), confirming that the channels are oriented along the axes of the individual cones constituting the aggregate.

**Figure 4:**
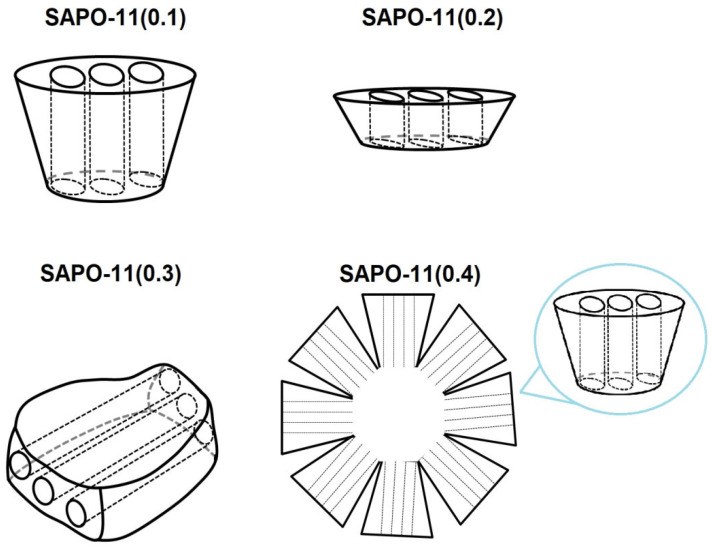
Schematic illustration of channel localization within SAPO-11 crystals synthesized at different SiO_2_/Al_2_O_3_ ratios.

Thus, the obtained results (schematically summarized in Figure 4) demonstrate that varying the silicon content in the reaction gel is a simple but effective method for the crystal engineering of SAPO-11. This approach allows for the deliberate manipulation of not only the crystal morphology (from 3D prisms and 2D platelets to complex hierarchical aggregates) but also, more importantly, for controlling the diffusion path length within the 1D channels.

As detailed TEM-SAED analysis revealed, the length of the 1D 10R channels in the synthesized samples varies widely: from ~200 nm (in the flat prisms of SAPO-11(0.2)) to ~100 nm (in the cones of SAPO-11(0.1)) and ~300 nm (in the prisms of SAPO-11(0.3)). In the case of the hierarchical aggregates (SAPO-11(0.4)), the channel mouths are distributed across the entire external surface of the spheres, which potentially enhances the accessibility of the active sites. This demonstrated ability to control crystal architecture at the nano- and mesoscale opens new avenues for optimizing the catalytic properties of these materials, especially in diffusion-limited reactions

**Figure 5:**
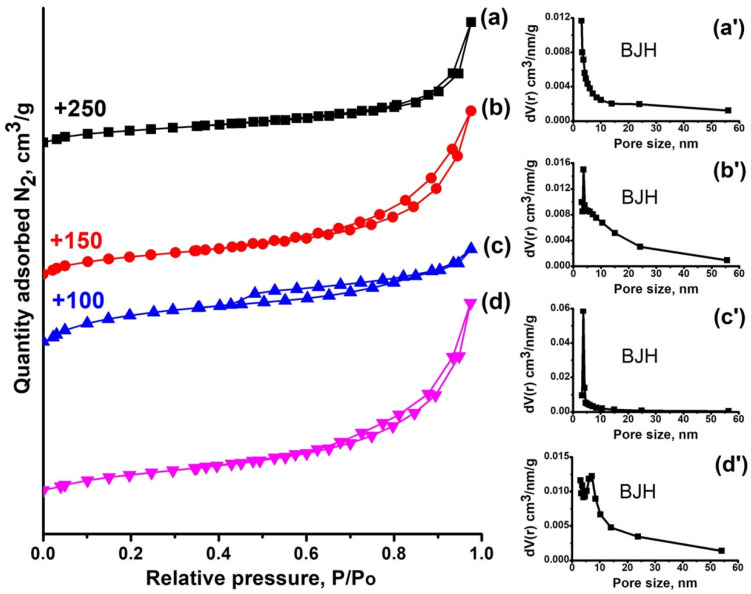
Nitrogen adsorption–desorption isotherms and pore size distribution of SAPO-11 samples synthesized at different SiO_2_/Al_2_O_3_ ratios: (**a**, **a’**) SAPO-11-0.1; (**b**, **b’**) SAPO-11-0.2; (**c**, **c’**) SAPO-11-0.3; (**d**, **d’**) SAPO-11-0.4.

The porous structure of the synthesized SAPO-11 samples was studied using nitrogen adsorption–desorption measurements. The isotherms and corresponding pore size distributions (PSD) are presented in Figure 5, and the key textural characteristics are summarized in Table 2. All materials exhibit Type IV isotherms with a distinct hysteresis loop, which is characteristic of hierarchical micro-mesoporous structures. PSD analysis confirms the presence of a broad mesopore distribution in the range of 2 to 50 nm. (It should be noted that the maximum in the region of ~2 nm is a known artifact associated with the tensile strength effect of nitrogen at low P/P_0_ and does not reflect real microporosity [31,32].)

The textural properties exhibit a clear dependency on the silicon content in the reaction gel. As the SiO_2_/Al_2_O_3_ ratio increases from 0.1 to 0.3, a growth in the specific surface area (S_BET_) and, particularly importantly, in the external surface area (S_EX_) is observed, which is likely due to the reduction in primary crystal size.

However, a further increase in the ratio to SiO_2_/Al_2_O_3_ = 0.4 leads to a noticeable decrease in both S_BET_ and S_EX_. This reduction can be explained by a combination of two factors: (1) decreased crystallinity (as will be shown later), which leads to the formation of a non-porous amorphous silicoaluminophosphate phase and reduces the micropore volume; (2) a change in morphology, specifically the formation of larger crystalline intergrowths, which reduces the accessible external surface area.

As the SiO_2_/Al_2_O_3_ ratio increases from 0.1 to 0.4, the micropore volume decreases steadily from 0.08 to 0.05 cm^3^·g^−1^. The largest mesopore volume, observed for the SAPO-11(0.4) sample, is associated with the formation of its specific secondary porous structure in the form of hierarchical aggregates composed of intergrown conical crystals (200–400 nm). The SAPO-11(0.2) sample characterized by a plate-like morphology also displays a remarkably high mesopore volume of 0.19 cm^3^·g^−1^, while retaining a micropore volume of 0.08 cm^3^·g^−1^, comparable to SAPO-11(0.1) (0.08 and 0.14 cm^3^·g^−1^ for micro- and mesopores, respectively). The SAPO-11(0.3) sample shows intermediate values of 0.06 and 0.14 cm^3^·g^−1^ for micro-- and mesopores, respectively.

It is well known that the catalytic properties of SAPO-n materials, particularly the strength and concentration of Brønsted acid sites (BAS), are determined by the mechanism of silicon (Si) incorporation into the aluminophosphate framework [12]. The established mechanisms are SM2 and SM2+SM3. The SM2 mechanism leads to the formation of “isolated” Si atoms in a Si(0Si, 4Al) environment, generating the maximum number of BAS. In contrast, the combination of SM2+SM3 results in the formation of “silicon islands”, where Si atoms in the core (with a Si(4Si, 0Al) environment) do not create acidity, and BAS are formed only at the boundaries of these islands (in environments such as Si(4−nSi, nAl), where *n* ≥ 1) [12,13].

**Figure 6:**
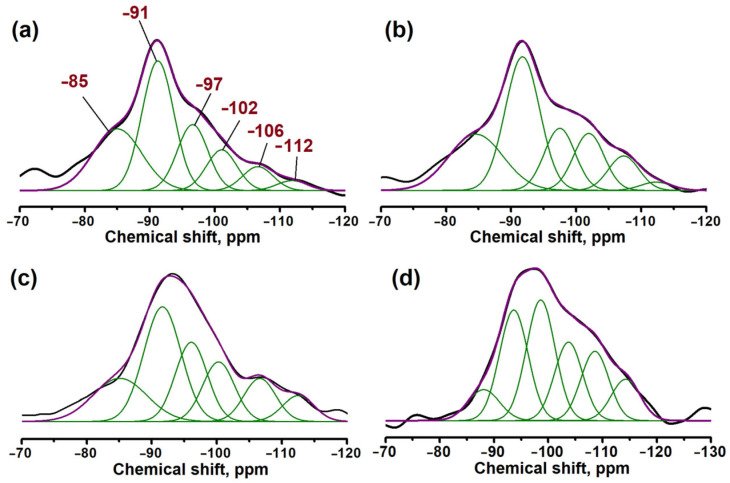
^29^Si Magic-Angle Spinning (MAS) NMR spectra of SAPO-11 samples synthesized at different SiO_2_/Al_2_O_3_ ratios: (**a**) SAPO-11(0.1); (**b**) SAPO-11(0.2); (**c**) SAPO-11(0.3); (**d**) SAPO-11(0.4).

To investigate the local environment of silicon atoms, ^29^Si MAS NMR spectroscopy was employed (Figure 6, Table 3). All samples exhibit a broad resonance in the chemical shift range from −85 to −112 ppm, indicating a heterogeneous distribution of Si environments. The spectra were deconvoluted into six Gaussian peaks. The signals at −91, −97, −102, −106, and −112 ppm are assigned to silicon atoms in the environments of Si(0Si, 4Al), Si(1Si, 3Al), Si(2Si, 2Al), Si(3Si, 1Al), and Si(4Si, 0Al), respectively [16,20]. An additional signal at −85 ppm is commonly attributed to silicon atoms within amorphous aluminosilicate fragments [16,20,33,34].

Analysis of the signal intensities (Table 3) revealed a direct correlation between the gel composition and the mechanism of Si incorporation. At low Si content (SAPO-11(0.1)), the highest total intensity of signals is observed in the range from −85 to −106 ppm. This indicates the dominance of the SM2 mechanism (peak at −91 ppm) and the formation of predominantly small silicon islands. Presumably, at low concentration, the initial SiO_2_ particles effectively depolymerize and incorporate into the framework as isolated atoms. With an increase in the SiO_2_/Al_2_O_3_ ratio, a shift in the distribution towards more shielded signals (from −102 to −112 ppm) is observed. For SAPO-11(0.4), the signals corresponding to large silicon islands (Si(2Si, 2Al)–Si(4Si, 0Al)) dominate, which clearly indicates the predominance of the SM2+SM3 incorporation mechanism [35,36].

**Figure 7:**
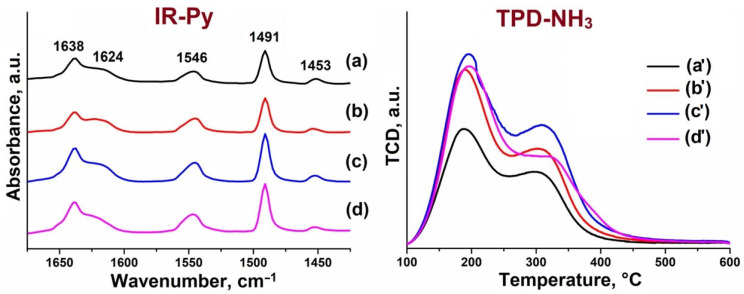
IR spectra of adsorbed pyridine (IR-Py) over SAPO-11 samples after desorption at 150 °C and ammonia temperature-programmed desorption (NH_3_-TPD) profiles: (**a**, **a’**) SAPO-11(0.1); (**b**, **b’**) SAPO-11(0.2); (**c**, **c’**) SAPO-11(0.3); (**d**, **d’**) SAPO-11(0.4).

The acidic properties of the samples, being a key factor in catalysis, were investigated using infrared spectroscopy of adsorbed pyridine (IR-Py) and temperature-programmed desorption of ammonia (NH_3_-TPD) (Figure 7, Table 4). The IR-Py spectra show characteristic absorption bands at 1545 cm^−1^ (pyridinium ion on Brønsted acid sites, BAS) and 1455 cm^−1^ (coordinated pyridine on Lewis acid sites, LAS). The band at 1490 cm^−1^ arises from contributions of both types of acid centers [37,38].

Quantitative analysis (Table 4) reveals a non-monotonic dependence of acidity on the silicon content. The concentration of both BAS and LAS increases with the SiO_2_/Al_2_O_3_ ratio, reaching a maximum at a value of 0.3. A further increase in the Si content (to 0.4) leads to a decrease in the concentration of acid sites. These results are in full agreement with the ^29^Si MAS NMR and XRD data. The initial increase in acidity (up to 0.3) is attributed to the rising number of isolated Si atoms (SM2 mechanism). The subsequent decrease (at 0.4) is explained by the two factors mentioned previously: (1) the predominance of the SM2+SM3 mechanism (formation of “silicon islands,” where only a small fraction of Si generates acidity) and (2) the decrease in sample crystallinity (formation of an amorphous phase lacking acid sites).

The NH_3_-TPD results (Table 4) correlate well with the IR-Py data, further confirming that the maximum total acidity is achieved at an SiO_2_/Al_2_O_3_ ratio of 0.3. Thus, increasing the silicon content in the reaction gels beyond 0.3 is ineffective for further enhancing the concentration of acid sites in the SAPO-11 structure.

The aforementioned results demonstrate that varying the silicon content in the reaction gel is a simple tool for simultaneously controlling the morphology, textural properties, and acidic characteristics of SAPO-11 materials. To evaluate the influence of these factors on catalysis, bifunctional Pt/SAPO-11 catalysts were prepared.

Bifunctional catalysts (~0.5 wt.% Pt) were prepared by impregnating the SAPO-11 supports with an H_2_PtCl_6_ solution using the incipient wetness method. The choice of Pt loading (~0.5 wt.%) is based on previous studies [37], which demonstrated that this metal loading provides sufficient hydrogenating–dehydrogenating function, thereby making the acid-catalyzed step on the support the rate-limiting stage of the hydroisomerization process. The platinum dispersion characteristics (Table 5) revealed a clear correlation with the textural properties of the supports. Samples with a more developed external surface area (S_EX_) and larger mesopore volume exhibited better Pt dispersion, as evidenced by smaller average particle sizes (in the range of 2.6–3.1 nm). This underscores the importance of the support’s secondary porous structure for stabilizing metal nanoparticles.

**Figure 8:**
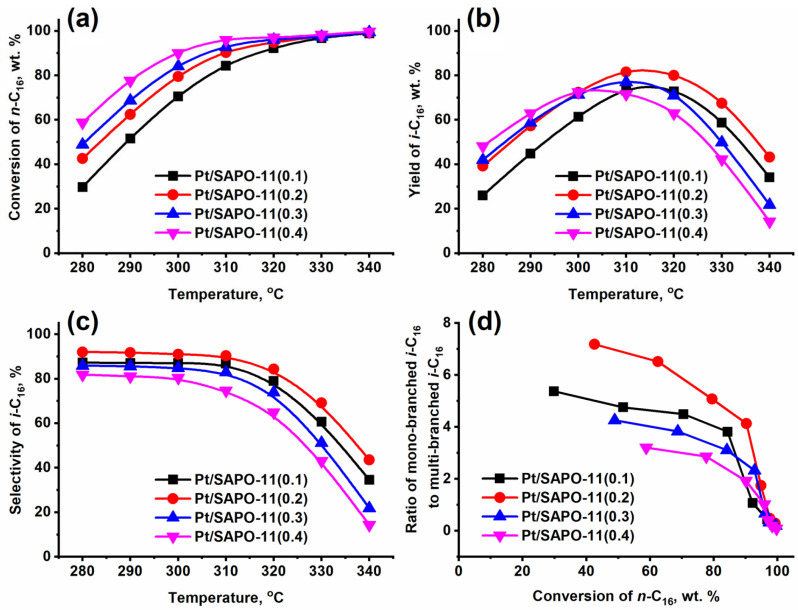
Hydroisomerization of *n*-hexadecane over 0.5 wt% Pt/SAPO-11 catalysts: (**a**) *n*-hexadecane conversion versus reaction temperature; (**b**) yield of C_16_ isomers versus reaction temperature; (**c**) selectivity to C_16_ isomers versus reaction temperature; (**d**) ratio of mono-branched to multi-branched C_16_ isomers versus *n*-hexadecane conversion.

The catalytic testing results for *n*-hexadecane hydroisomerization are presented in Figure 8 and Table 6. It was found that the main reaction products are mono- and di-methyl substituted C_16_ isomers, which is consistent with the “pore mouth” catalysis mechanism. As expected, increasing the reaction temperature leads to a rise in *n*-hexadecane conversion but is accompanied by a decrease in selectivity towards C_16_ isomers due to enhanced side reactions of hydrocracking. Consequently, the yield of C_16_ isomers (*i*-C_16_) passes through a maximum at 310–320 °C.

Figure 8, which shows the change in conversion as a function of temperature, shows that with increasing silicon content and, consequently, with increasing acid site concentration, especially strong Brønsted acid sites, the overall activity in *n*-hexadecane hydroconversion increases across the entire Pt/SAPO-11 catalyst series.

Notably, despite an acid site density comparable to or even slightly lower than SAPO-11(0.3), the Pt/SAPO-11(0.4) catalyst exhibits the highest activity, which is presumably attributed to a significantly larger mesopore volume, which can increase the effective concentration of active sites accessible to reactant molecules.

The catalyst Pt/SAPO-11(0.2) provides the maximum selectivity towards C_16_ isomers and the highest *i*-C_16_ yield (reaching 81%). We attribute this result to the unique morphology of the SAPO-11(0.2) support. This sample is characterized by flat prismatic crystals with the shortest length of the 1D 10R channels (~80 nm). This morphology minimizes the residence time of intermediates within the micropores. This effectively suppresses secondary reactions (subsequent cracking) and enhances the selectivity towards the target mono-branched products. Consistent with this, the highest C_16_ isomer yields were observed for Pt/SAPO-11(0.2) (81%), followed by Pt/SAPO-11(0.3) (77%), Pt/SAPO-11(0.1) (73%), and Pt/SAPO-11(0.4) (71%).

Moreover, analysis of product distribution reveals that the ratio of mono-branched to multi-branched C_16_ isomers is consistently highest over Pt/SAPO-11(0.2) at all conversion levels. This behavior is likely a consequence of its optimal combination of moderate acidity and plate-like crystal morphology, which enables rapid diffusion of *n*-hexadecane and its isomers, thereby limiting the opportunity for further isomerization beyond the mono-branched stage.

In contrast, the Pt/SAPO-11(0.4) sample, which possesses a higher concentration of acid sites compared to Pt/SAPO-11(0.2), exhibits significantly worse selectivity. This is directly related to its morphology (large crystals and intergrowths), which creates severe diffusion limitations. The prolonged contact time of reactants with active sites inside the pores leads to the dominance of undesirable hydrocracking reactions, drastically reducing the yield of the target isomers. Similarly, Pt/SAPO-11(0.1) and Pt/SAPO-11(0.3), both featuring larger crystal sizes than Pt/SAPO-11(0.2), also display lower isomer selectivity.

The most pronounced loss of selectivity is observed for Pt/SAPO-11(0.4), which not only has the largest crystal dimensions but also the strongest acidity, promoting excessive branching and, consequently, higher yields of cracked by-products alongside multi-branched isomers.

**Figure 9:**
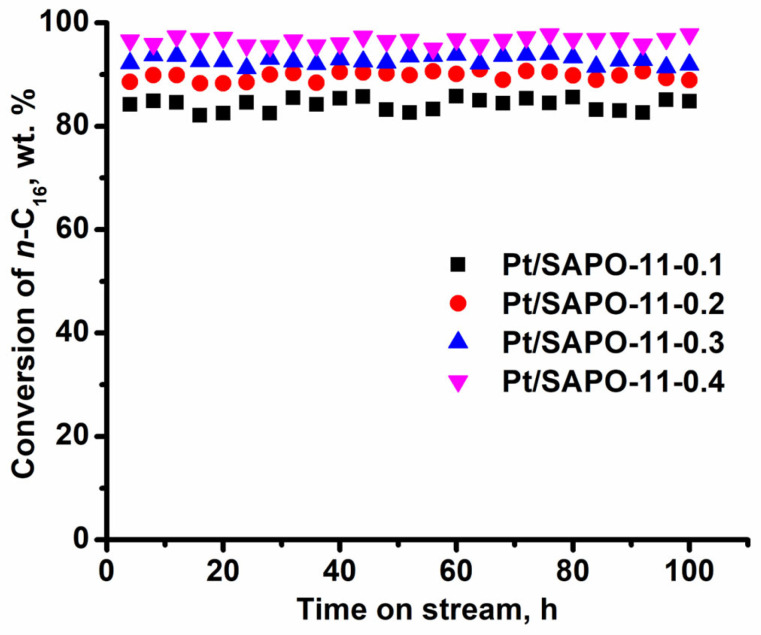
Hydroisomerization of *n*-hexadecane over 0.5 wt% Pt/SAPO-11 catalysts at 3.0 MPa, 310 °C, WHSV = 2.0 h^−1^, and H_2_/*n*-C_16_ = 10 mol/mol for 100 h.

Catalyst stability was evaluated by conducting a 100-h time-on-stream test under standard hydroisomerization conditions using pure *n*-hexadecane feed (free of sulfur-, nitrogen-containing and aromatic compounds) and a high hydrogen pressure. No significant deactivation was observed for any of the Pt/SAPO-11 catalysts over the entire test duration, as evidenced by stable *n*-hexadecane conversion (Figure 9).

## 3. Conclusions

This work demonstrates that varying the molar SiO_2_/Al_2_O_3_ ratio in the initial boehmite-based reaction gel serves as a simple, accessible but effective tool for the comprehensive control of the physicochemical properties of a SAPO-11 molecular sieve. It has been established that this parameter enables control not only over the phase composition and crystallinity but also over the silicon incorporation mechanism; the properties of the porous structure; and, most importantly, the crystal morphology and size.

It was shown that the key material characteristics exhibit a clear dependence on the initial silicon content. The specific surface area (S_BET_), external surface area (S_EX_), and concentration of Brønsted acid sites (BASs) reach their maximum values at an SiO_2_/Al_2_O_3_ ratio of 0.3. A further increase in the Si content (SiO_2_/Al_2_O_3_) to 0.4 does not lead to an increase in acidity but, on the contrary, causes a noticeable decrease in crystallinity and a reduction in the concentration of acid sites. The ^29^Si MAS NMR data convincingly demonstrated that this is due to reaching the incorporation limit for the SM2 mechanism (which generates BASs) and a transition to the dominant SM2+SM3 mechanism, leading to the formation of large “silicon islands” that do not generate acidity.

Catalytic testing in the hydroisomerization of n-hexadecane revealed a complex relationship between the support properties and its performance. For all investigated samples, a dependency was observed where *n*-hexadecane conversion increases and selectivity towards C_16_ isomers decreases with rising temperature.

A key finding of this study is that the highest selectivity and maximum yield of the target C_16_ isomers (reaching 81%) were achieved with the Pt/SAPO-11(0.2) catalyst. This sample, while not possessing the maximum acidity, is characterized by a unique platelet-like prism morphology. As confirmed by TEM-SAED analysis, this architecture ensures the shortest diffusion path length (~100 nm) along the 1D channels within the series. It is precisely this structural advantage that minimizes the residence time of intermediates within the pores, effectively suppressing undesirable secondary hydrocracking reactions. In contrast, samples with longer diffusion paths (e.g., Pt/SAPO-11(0.4)) exhibited significantly worse selectivity due to enhanced diffusion limitations.

Thus, this work demonstrates that for developing highly selective bifunctional hydroisomerization catalysts based on SAPO-11, optimizing morphology and minimizing diffusion path length are more critical factors than simply achieving the maximum concentration of acid sites. This conclusion opens new perspectives for the rational design of SAPO-n catalysts through targeted crystal engineering.

## 4. Materials and Methods

### 4.1. Synthesis of SAPO-11

Silicoaluminophosphate SAPO-11 was synthesized by hydrothermal crystallization. The following starting reagents were used: orthophosphoric acid (H_3_PO_4_, 85 wt%, Reakhim, Russia) as the phosphorus source, boehmite (AlOOH) as the aluminum source, and a SiO_2_ sol (prepared according to [26]) as the silicon source. Di-*n*-propylamine (DPA, 99 wt%, Acros Organics, Schwerte, Germany) was used as the structure-directing agent (template), and distilled water served as the solvent.

A series of initial reaction gels with varying silicon content was prepared, having the final molar composition: 1.0Al_2_O_3_•1.0P_2_O_5_•x SiO_2_•1.0DPA•40H_2_O, where the molar ratio SiO_2_/Al_2_O_3_ took values of 0.0, 0.1, 0.2, 0.3, and 0.4. A typical synthesis procedure was as follows: 10.0 g of H_3_PO_4_ (85%) was dissolved in 28.0 g of distilled water. To the resulting solution, 5.6 g of boehmite was added portionwise under vigorous stirring until a homogeneous suspension (aluminophosphate gel) formed. Immediately after this, a calculated amount of SiO_2_ sol, corresponding to the target SiO_2_/Al_2_O_3_ ratio, was introduced into the aluminophosphate gel.

The resulting reaction gel was stirred vigorously for 1 h to achieve homogeneity, followed by aging under static conditions in a thermostat at 90 °C for 24 h. The aged gel was transferred to a stainless-steel autoclave with a Teflon liner, and hydrothermal crystallization was carried out under static conditions at 200 °C for 24 h. Upon completion of the synthesis, the autoclave was cooled to room temperature.

The solid product was separated by centrifugation, repeatedly washed with distilled water until the washings reached neutral pH, and finally dried in an oven at 90 °C for 24 h. The initial gel mixtures were designated as SAPO-(x), and the final crystalline products after synthesis as SAPO-11(x), where x represents the molar SiO_2_/Al_2_O_3_ ratio.

### 4.2. Material Analysis Methods

The elemental composition of the silicoaluminophosphate gels, crystalline products and platinum-containing samples was determined by X-ray fluorescence (XRF) spectroscopy using a Shimadzu EDX-7000P (Shimadzu Corporation, Duisburg, Germany) spectrometer and the fundamental parameters method.

Powder X-ray diffraction (XRD) patterns of the as-synthesized (uncalcined) SAPO-11 samples were recorded on a Shimadzu XRD-7000 diffractometer (Shimadzu Corporation, Kyoto, Japan), CuKα radiation, λ = 1.5406 Å. Scanning was performed in the 2θ range from 5 to 40° at a rate of 1°/min. Phase identification was carried out using the PDF-2 database (version 2.2201). The degree of crystallinity was estimated as the ratio of the integrated intensities of the crystalline peaks to the total area (including the amorphous halo) in the 2Ɵ range of 20–30° using the Shimadzu XRD Crystallinity software (Japan, version 7.04). To account for the uncertainty in baseline fitting in the presence of prominent Bragg reflections, the estimated error of the crystallinity determination is ± 2%.

Target-oriented approach was utilized for the optimization of the analytic measurements [38]. Before measurements, the samples were deposited on the 3 mm carbon-coated copper grids from isopropanol suspension. The observations were carried out using Hitachi Regulus SU8230 field-emission scanning electron microscope (Hitachi High-Tech Corporation, Tokyo, Japan). Images were acquired in transmitted electron mode at 30 kV accelerating voltage.

The microstructure of SAPO-11 samples was examined by transmission electron microscopy (TEM) using a Hitachi HT7700 electron microscope (Hitachi High-Tech Corporation, Tokyo, Japan). Images were acquired in bright-field mode at an accelerating voltage of 100 kV. Detailed structural analysis of the final molecular sieves was performed using a high-resolution transmission electron microscope (HRTEM), ThemisZ (Thermo Fisher Scientific, Waltham, MA, USA). The analysis was carried out at an accelerating voltage of 200 kV, with the instrument achieving a maximum lattice resolution of 0.07 nm. Images were recorded using a Ceta 16 CCD camera (Thermo Fisher Scientific, Waltham, MA, USA). Sample preparation was conducted as follows: the material was dispersed in ethanol by ultrasonication, and the resulting suspension was deposited onto copper grids coated with a perforated carbon film. Image processing for interplanar spacing calculations was performed by fast Fourier transform (FFT) using the Digital Micrograph software package (Gatan, Inc., Pleasanton, CA, USA, version 3.8).

Textural properties (specific surface area, micro- and mesopore volumes) were measured by low-temperature (−196 °C) nitrogen adsorption–desorption using an Altamira Instruments QUICK-200 analyzer (Altamira Instruments Co., Beijing, China). The specific surface area was calculated using the multi-point Brunauer–Emmett–Teller (BET) method. The micropore volume was evaluated using the t-plot method. The pore size distribution was calculated using the Barrett-Joyner-Halenda (BJH) model applied to the desorption branch of the isotherm.

^29^Si CP/MAS NMR spectra were recorded on a Bruker Avance-400 (Bruker Corporation, Billerica, MA, USA) spectrometer (operating frequency: 79.49 MHz for ^29^Si) using a 4 mm H/X MAS probe. Cross-polarization (CP/MAS) with a RAMP sequence (70–100% proton ramping) and a contact time of 3 ms was applied. SW-TPPM decoupling (8 μs, 15°) was used to suppress proton splitting. A 90° 1H pulse of 2.5 μs duration and a relaxation delay of 10 s were employed. The accumulated number of scans per spectrum ranged from 8000 to 13,000 at a magic-angle spinning (MAS) rate of 8 kHz. All chemical shifts are reported relative to tetramethylsilane (TMS) at 0 ppm. The resulting spectra were deconvoluted using the Dmfit software package (version 1.0).

The total acidity of the samples and the strength distribution of acid sites were studied by ammonia temperature-programmed desorption (NH_3_-TPD) using an Altamira AMI-400TPx setup (Altamira Instruments Co., Beijing, China). Prior to analysis, the sample (approximately 100 mg) was calcined in situ in a helium flow (30 mL/min) at 600 °C for 4 h. The temperature was then lowered to 100 °C, and the sample was saturated with a gas mixture (10 vol% NH_3_ in helium) for 30 min. Physically adsorbed ammonia was removed by purging with helium at 100 °C until the baseline stabilized. Ammonia desorption was monitored using a thermal conductivity detector (TCD) in the temperature range of 100–600 °C with a heating rate of 10 °C/min.

The types of acid sites (Brønsted, BAS, and Lewis, LAS) and their concentrations were determined using Fourier-transform infrared spectroscopy of adsorbed pyridine (IR-Py). Spectra were recorded on a Bruker Vertex-70V spectrometer (Bruker Optic GmbH, Ettlingen, Germany) with a resolution of 4 cm^−1^ in the range of 4000–400 cm^−1^. Tableted samples (10 mg/cm^2^) were prepared and pre-treated by calcination under vacuum (450 °C, 1 h). Pyridine adsorption was carried out at 150 °C for 30 min, after which physically adsorbed pyridine was removed by evacuation at the same temperature for 30 min. The concentrations of BAS and LAS were calculated by integrating the absorption bands at 1545 cm^−1^ (pyridinium ion) and 1454 cm^−1^ (coordinated pyridine), respectively, using the corresponding molar extinction coefficients [39].

Hydrogen pulse chemisorption was used to determine the platinum dispersion and average particle size in pre-reduced catalysts. The analysis was performed on an Altamira AMI-400TPx unit (Altamira Instruments Co., Beijing, China). An adsorption stoichiometry of H/Pt = 1 was assumed for the calculations.

### 4.3. Preparation of Pt/SAPO-11 Catalysts

Pt-containing catalysts (0.5 wt% Pt/SAPO-11) were prepared by the incipient wetness impregnation method. The support material (SAPO-11, 0.1–0.5 mm fraction), previously calcined in air at 600 °C for 6 h, was impregnated with an aqueous solution of hexachloroplatinic acid (H_2_PtCl_6_•6H_2_O, Acros Organics, Morris Plains, NJ, USA) of appropriate concentration. The impregnated samples were then dried at 100 °C for 24 h and calcined in air at 550 °C for 5 h. The calcined catalysts were pelletized, crushed, and sieved to obtain the working fraction of 0.1–0.2 mm.

### 4.4. Determination of Platinum Dispersion

Platinum dispersion and average particle size were determined by hydrogen pulse chemisorption using an Altamira AMI-400TPx apparatus (Altamira Instruments Co., Beijing, China). Prior to analysis, the samples were reduced in situ under a H_2_ flow (50 mL/min) at 400 °C for 2 h. Measurements were conducted at 40 °C. A chemisorption stoichiometry of H:Pt = 1 was assumed for the calculations.

### 4.5. n-Hexadecane Hydroisomerization

Catalytic testing was performed in a stainless-steel fixed-bed flow reactor (inner diameter 10 mm) at a pressure of 3.0 MPa and temperatures ranging from 280 to 350 °C. Prior to the reaction, the catalyst (approximately 1 g) was reduced in situ under hydrogen flow at 400 °C and 3.0 MPa for 5 h. The reaction was conducted with a molar H_2_/*n*-C_16_ ratio of 12 and a weight hourly space velocity (WHSV) of 2 h^−1^.

Product analysis was performed online. Gaseous products were analyzed by gas chromatography (GC) using an Chromatek-Crystal 5000 chromatograph (Chromatec, Yoshkar-Ola, Russia) equipped with a flame ionization detector and a capillary column (HP-1,50 m). Liquid products were identified by gas chromatography-mass spectrometry (GC-MS) using a Shimadzu GCMS-TQ8050 NX instrument (Shimadzu Corporation, Kyoto, Japan).

## Figures and Tables

**Table 1:** Phase composition and chemical analysis of SAPO-11 molecular sieves synthesized at different initial SiO_2_/Al_2_O_3_ ratios.

Sample	Chemical Composition, Gel Al_2_O_3_●P_2_O_5_●SiO_2_	Chemical Composition, AEL Al_2_O_3_●P_2_O_5_●SiO_2_	Phase	Degree of Crystallinity, %
SAPO-11-0.1	1.00:1.00:0.10	1.00:0.98:0.06	AEL	94
SAPO-11-0.2	1.00:1.00:0.20	1.00:0.95:0.12	AEL	93
SAPO-11-0.3	1.00:1.00:0.30	1.00:0.92:0.20	AEL	90
SAPO-11-0.4	1.00:1.00:0.40	1.00:0.91:0.22	AEL	81

**Table 2:** Textural properties of SAPO-11 molecular sieves synthesized at different SiO_2_/Al_2_O_3_ ratios.

Sample	S_BET_, m^2^/g	S_EX_, m^2^/g	V_micro_, cm^3^/g	V_meso_, cm^3^/g
SAPO-11(0.1)	190	46	0.08	0.14
SAPO-11(0.2)	223	67	0.08	0.19
SAPO-11(0.3)	283	118	0.06	0.14
SAPO-11(0.4)	232	87	0.05	0.24

S_BET_—specific surface according to BET. S_EX_—external specific surface area. V_micro_—specific volume of micropores. V_meso_—specific volume of mesopores.

**Table 3:** Deconvolution results of ^26^Si MAS NMR spectra for SAPO-11 synthesized at different SiO_2_/Al_2_O_3_ ratios.

Sample	Deconvolution, (%)					
	−85 ppm	Si (0Si, 4Al) −91 ppm	Si (1Si, 3Al) −97 ppm	Si (2Si,2Al) −102 ppm	Si (3Si, 1Al), −107 ppm	Si (4Si, 0Al), −112 ppm
SAPO-11(0.1)	26	37	19	10	6	2
SAPO-11(0.2)	24	36	15	13	9	3
SAPO-11(0.3)	17	32	19	14	11	7
SAPO-11(0.4)	7	24	26	17	16	10

**Table 4:** Acid properties of SAPO-11 supports (NH_3_-TPD, IR spectroscopy of adsorbed pyridine).

Sample	Acid Sites Concentration by NH_3_–TPD, μmol/g			Acid Sites Concentration by IR Spectra of Adsorbed Pyridine, μmol/g	
	«Weak» ^a^	«Moderate» ^b^	«Strong» ^c^	BAS	LAS
SAPO-11(0.1)	141	126	8	87	18
SAPO-11(0.2)	219	176	18	106	12
SAPO-11(0.3)	249	201	30	145	20
SAPO-11(0.4)	234	180	33	141	15

^a^ the amount of ammonia desorbed in the range of 100–250 °C; ^b^ the amount of ammonia desorbed in the range of 250–400 °C; ^c^ the amount of ammonia desorbed in the range of 400–600 °C; BAS—Brønsted acid sites; LAS—Lewis acid sites.

**Table 5:** Pt content and particle size of Pt in Pt/SAPO-11.

Sample	Pt Content ^a^, wt.%	Pt particle Size ^b^, nm	Pt/H^+^
Pt/SAPO-11(0.1)	0.48	3.7	0.078
Pt/SAPO-11(0.2)	0.52	3.3	0.073
Pt/SAPO-11(0.3)	0.47	2.6	0.067
Pt/SAPO-11(0.4)	0.45	3.1	0.058

^a^ determined by XRF, ^b^ particle size of platinum obtained by pulse chemisorption method; Pt/H^+^—The ratio of the number of metal sites Pt obtained by pulse chemisorption method to the concentration of BAS.

**Table 6:** Results of *n*-C_16_ hydroisomerization over Pt/SAPO-11 at 3.0 MPa, 310 °C, WHSV = 2.0 h^−1^, and H_2_/*n*-C_16_ = 10 mol/mol.

	Pt/SAPO-11- 0.1	Pt/SAPO-11- 0.2	Pt/SAPO-11-0.3	Pt/SAPO-11-0.4
Conversion, wt. %	84.3	90.3	92.8	95.9
S_∑_^a^, %	86.8	90.3	83.0	74.7
S_MB_^a^, %	68.8	72.7	58.1	37.9
S_MTB_^a^, %	18.0	17.6	24.9	36.8
3-mC_15_, wt. %	9.6	10.6	8.4	5.3
2-mC_15_, wt. %	9.9	11.0	7.8	4.9
4-mC_15_, wt. %	7.4	8.5	7.5	5.2
5-mC_15_, wt. %	4.9	7.3	7.5	4.5
∑6–8-mC_15_, wt. %	26.2	28.2	22.7	16.4
MTB-C_16_^b^, wt. %	15.2	15.9	23.1	35.3

^a^ S_∑_, S_MB_, and S_MTB_ correspond to the selectivity of total C_16_H_34_ isomers, monobranched C_16_H_34_ isomers, and multibranched C_16_H_34_ isomers, respectively. ^b^ MTB-C_16_ denotes the multi-branched *i*-C_16_ isomers.

## Data Availability

The original contributions presented in the study are included in the article; further inquiries can be directed to the corresponding authors.
